# Prevalence and determinants of condom use among the youth in Malawi: evidence from the 2015/16 Malawi Demographic and Health Survey

**DOI:** 10.1186/s12978-023-01714-9

**Published:** 2023-11-21

**Authors:** Scholastica Eunice Jimu, Lorretta F. C. Ntoimo, Friday E. Okonofua

**Affiliations:** 1 Centre of Excellence in Reproductive Health Innovation, University of Benin, Benin, Nigeria; 2 Department of Demography and Social Statistics, Faculty of Social Sciences, Federal University Oye-Ekiti, Oye Ekiti, Nigeria; 3 Obstetrics and Gynecology, and Reproductive Health, Centre of Excellence in Reproductive Health Innovation (CERHI), World Bank Projects, University of Benin, Benin, Nigeria; 4 Women’s Health and Action Research Centre (WHARC), Benin, Nigeria; 5 African Journal of Reproductive Health (AJRH), Benin, Nigeria

**Keywords:** Condom use, Sexually transmitted infections, HIV, Unplanned pregnancy, Youth

## Abstract

**Background:**

Unprotected sexual intercourse among the youth is common in Malawi. This has led to high rates of sexually transmitted infections (STIs), Human Immunodeficiency Virus (HIV), and unplanned pregnancies. The study investigated the prevalence and the determinants of male and female condom use for the prevention of sexually transmitted infections and unplanned pregnancies among the youth in Malawi.

**Methods:**

The 2015/2016 Malawi Demographic and Health Survey (MDHS) data were used among 15 to 24-year-old male and female who had sexual intercourse four months preceding the survey. A total of 1543 male and 5143 female were selected from 3226 male and 10,367 female respectively and analyzed with SPSS version 20 using.descriptive, bivariate, and logistic regression.

**Results:**

The study found a low prevalence (27.1%) of condom use among the youth in the last sexual intercourse within four months before the survey. More male (55.8%) used condoms than female (18.5%). The significant predictors of condom use among the male and female youth were: being sexually active (OR 0.39 CI 0.33–0.47), aged 20–24 (OR 0.80 CI 0.68–0.95), ever married (OR 0.07 CI 0.06–0.08), coming from central region (OR 0.56 CI 0.40–0.77), and southern region (OR 0.59 CI 0.42–0.83), residing in the rural area (OR 0.74 CI 0.61–0.90) and ever tested of HIV (OR 1.29 CI 1.03–1.55).

**Conclusion:**

Having established low prevalence of condom use among the youth in Malawi, there is a need to scale up programs and policies that target the youth to practice safe sex, which will assist in addressing the challenges of STIs, including HIV, and preventing unplanned pregnancies in Malawi**.**

## Background

Male and female condom use is critical for comprehensive and sustainable prevention of sexually transmitted infections (STIs), human immunodeficiency virus (HIV), and unplanned pregnancies, in a comprehensive and long-term way [1–5]. UNAIDS reported that, condom use has prevented approximately 50 million new HIV infections worldwide since the onset of the epidemic [6, 7]. An estimation of 27 billion condoms were anticipated to be distributed globally in 2015 to protect about 225 million couples from unplanned pregnancies [6]. In addition, the 2016–2021 UNAIDS Strategy aimed at increasing the availability of condoms to 20 billion per year by 2020 in low- and middle-income countries [7]. However, condom use among the high risk population (15–49 years) throughout the world remains low [6, 8, 9]. UNAIDS reported a decline of condom use among men (15–49 years) and young women (15–24 years) across five countries in western and central Africa and three countries in eastern and southern Africa”[8]. In Malawi condom use among the youth with non-regular partner is between 53 to 73% with lowest use among female [10]. This has contributed to the estimated 3.9 million youth living with HIV throughout the world, of which about 84% are living in sub-Saharan Africa [6, 9]. Malawi registhird of 38,000 new HIV infections among the youth in 2018 [6]. Approximately 10 million unintended pregnancies have been reported to occur every year in the developing world among adolescent girls [11]..

Incidences of high-risk sexual behavior among youth are alarming. Understanding why youth practice unprotected sex which puts them at high risk of HIV and other sexually transmitted infections, as well as unplanned pregnancies is vital in addressing this public health challenge [1, 12]. Youth undergo a transition phase from childhood to adulthood and this is accompanied by several behavioral and developmental transformations which may influence them to explore their sexuality [13, 14].Several studies have been conducted in the Western world and Africa related to the prevalence and determinants of condom use among the youth [8, 12–16]. A study among African American and Hispanic/ Latino youth found that protected sex was higher among male than female and lower among gays/lesbians than heterosexuals [15]. Another study in Malawi found that approximately 28.7 percent of male and 35.6 percent of female using condoms inconsistently [16]. Some determinants of condom use among the youth include age, sex, place of residence, drug and alcohol abuse, duration of the relationship, perceived confidence to use a condom, perceived susceptibility to STIs and pregnancy, refusing to have sex if a partner refuses to use a condom, intention to bargain over the use of condoms, religion, relationship intimacy, sex-stigma and accessibility to condoms [17–19].

The Malawi’s 2015/2020 national condom strategy aims at improving the availability of and access to quality male and female condoms by all sexually active persons [20]. The policy recommends awareness and use of primary preventive measures to prevention of HIV infection, STIs, and unintended pregnancies in Malawi [20]. The Malawi Government and other stakeholders provides male and female condoms. For instance a rebranded *Chishango* male condom can be purchased at the market at a low cost of about K100 in a pack of 3 which is approximately to 0.13 United States dollars [21]. However, despite male and female condoms being affordable and only method that provides dual protection against unplanned pregnancies and STI’s including HIV but their use remains law [20]. Malawi’s condom distribution per capita is about four condoms per person per year [22] which are not enough to avert the countries demand In resource-constrained countries like Malawi with the majority of the population comprising of the youth, primary prevention is the best strategy to address public health concerns. A literature gap exists in Malawi regarding condom use among a broader population, as existing studies predominantly focus on commercial sex workers, married adults, and HIV-infected individuals, while studies involving college students and sexually active youths lack generalizability and comprehensive determinants of condom use [16, 23–26]. Therefore this study envisaged to contribute to existing body of knowledge by providing evidence-based information on the state of condom use and factors which can hinder or promote its use among the youth in Malawi using a national represented survey (MDHS). These will inform targeted interventions and strengthening the design and implementation of appropriate policies and programs to increase condom use among sexually active youth in Malawi.

The objective of this study was to determine the prevalence and determinants for condom use among male and female youth aged 15–24 years in Malawi.

## Methods and measures

### Study design and data source

This was a cross-sectional study that used archival data for male and female youth aged 15 to 24 who had sexual intercourse within the four months preceding the study. The archival data were extracted from the 2015–2016 Malawi Demographic Health Survey (MDHS)[27]. The archival data was downloaded free from https://www.dhsprogram.com/data/dataset_admin/login_main.cfm after obtaining permission from ICF Micro International. The MDHS is a nationally representative survey that draws respondents from households in rural and urban areas using multi-stage sampling design. To obtain a sample that comprised both male and female, the male recode and the individual woman recode of the MDHS were pooled, and male and female who were aged 25 and above were dropped.

### Study population and sample size

The study targeted male and female youth aged 15 to 24 in Malawi who have had sex 4 months preceding the study. A total of 1543 male and 5143 female met this inclusion criteria from a total of 3226 male and 10,367 female in the survey.

### Variables and measures

Condom use was the outcome variable. The respondents who used a condom four months prior to the survey were coded as 1, and those who did not use a condom were coded as 0. In addition, the independent variables of the study were extracted from the MDHS 2015/16 which included Age, sex, marital status, education level, ethnicity, wealth, religion, place of residence, number of sexual partners, working status, ever tested for HIV/AIDS: Sex was identified as male/female, age group was stratified into two groups 15–19 and 20–24, marital status was grouped into never married and ever married, place of residence was grouped into rural/urban, education status was defined as those with no education, primary, secondary/ higher, religion was grouped into Catholic, Protestant, Muslim/other, ethnicity was defined as Chewa, Tumbuka, Lomwe, Tonga, Yao, Sena, Nkhonde, Ngoni, Mang’anja, Nyanja and Others, region was defined as northern, central and southern, currently working was defined as yes (1)/ no (0), lifetime sexual partners was defined as 1 (those with one sexual partner), 2 (those with multiple sexual partner) and ever tested for HIV was identified as 0 (for no) and 1 (for yes).

### Data analysis

Data were analyzed using “Statistical Package for Social Science (SPSS)” version 20 and Microsoft excel for plotting graphs. The prevalence of condom use among the youth in Malawi was analyzed using frequencies and percentages. To identify the determinants of condom use among the youth in Malawi, chi-square and logistic regression were used.

## Results

### Sexual activity by youth in Malawi

Results in Table 1 shows that the majority of the youth 13,965(70.9%) ever had sex. In terms of sex, more female 10,367 (71.9%) than male 3226 (67.5%) have ever had sex. For those who never had sex, male were 1047 (32.5%) and female were 2918 (28.1%).

**Table 1:** Population of youth who had and never had sex. Source: Malawi Demographic and Health Survey (2015/16), weighted cases

Sex of respondents	Youth who never had sex	Youth who had sex
	n (%)	n (%)
All youth	3965 (29.1)	13, 593 (70.9)
Male	1047 (32.5)	3226 (67.5)
Female	2918 (28.1)	10,367 (71.9)

### Youth who had sex for the past 4 months before the study

Table 2 shows that out of 13,593 youth who have ever had sex, 6686 (49.2%) had sex within 4 months before the survey. In terms of sex, female accounted for 5143 (49.6%) while male was1, 543 (47.8%).

**Table 2:** Number of the youth who have ever had sex and those who had sex the past 4 months before the study. Source: Malawi Demographic and Health Survey (2015/16) survey data

Sex of respondents	Whoever had sex	Had sex last 4 months
	n	n (%)
All youth	13,593	6686 (49.2)
Male	3226	1543 (47.8)
Female	10,376	5143 (49.6)

### Socio-demographic characteristics of the study population

**Fig. 1:**
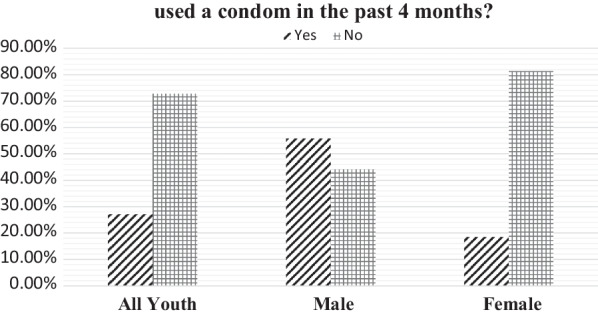
Youth who used a condom in the past 4 months? Source: Malawi Demographic and Health Survey (2015/16), survey data

Table 3 shows the socio-demographic characteristics of respondents. There were more female (5143**)** than male (1543); 61.4% of the male were aged 20–24; while 67.2% of the female were within the same age range. The majority (70.4%) of male and female youth were married. In terms of sex, never married male were in majority (64.2%) while more female (70.4%) were ever married. Most of the youth had more than one sexual partner, the majority of male had more than one sexual partner while female had one sexual partner. About 80.1% of the male and female youth were residing in the rural area and for the majority of them; their highest level of education was primary school. Most of the male and female respondents were affiliated with Protestants, Chewa ethnic group, and from the southern region of Malawi. 77.3% of male and 56.4% of the female were working and the majority of the male and female youth have ever tested for HIV (Fig. 1).

**Table 3:** Socio-demographic characteristics of the youth. Source: Malawi Demographic and Health Survey (2015/16), survey data

Characteristic	Male		Female		Youth	
	n = 1543	%	n = 5143	%	n = 6686	%
Age grouped						
15–19	594	38.5	1686	32.8	2280	34.1
20–24	949	61.5	3457	67.2	4406	65.9
Marital status						
Never married	991	64.2	986	19.2	1977	29.6
Ever married	552	35.8	4157	80.8	4709	70.4
Place of residence						
Urban	307	19.9	1026	19.9	1333	19.9
Rural	1236	80.1	4117	80.1	5353	80.1
Education						
No education	38	2.5	243	4.7	281	4.2
Primary	939	60.9	3389	65.9	4328	64.7
Secondary and higher	566	36.7	1511	29.4	2077	31.1
Religion						
Catholic	305	19.8	880	17.1	1185	17.7
Protestants	1028	66.8	3634	70.7	4662	69.7
Muslim and others	210	13.6	629	12.2	839	12.5
Ethnic group						
Chewa	494	32.0	1636	31.8	2130	31.9
Tumbuka	145	9.4	503	9.8	648	9.7
Lomwe	276	17.9	964	18.7	1240	18.5
Tonga	64	4.1	177	3.4	241	3.6
Yao	172	11.1	608	11.8	780	11.7
Sena	77	5.0	228	4.4	305	4.6
Nkhonde	29	1.9	62	1.2	91	1.4
Ngoni	195	12.6	623	12.1	818	12.2
Mang’anja	38	2.5	104	2.0	142	2.1
Nyanya	14	0.9	96	1.9	110	1.6
Others	39	2.5	142	2.8	181	2.7

### Prevalence of condom use among the youths in Malawi

The prevalence of condom use during the last sexual intercourse within 4 months before study among all the youth was 1813 (27.1%). In terms of sex, condom use among the male during the last sexual intercourse within 4 months before the study was higher 861 (55.8%) than that of female 952 (18.5%).

### Determinants of condom use among the youth in Malawi

Table 4: presents the determinants of condom use among the youth. Youth aged 15–19 (50.3%) used condoms slightly above those who were 20–24 years(49.7%). never married youth (75.4%) used condoms more than ever married youth (24.6%), most (73.1%) youth residing in the rural areas used condoms more than 26.9% from urban areas, Slightly most (54.2%) of the youth who had attended at least primary school used condoms more than who had no education and those with secondary and higher education, the proportion of youth from chewa ethnic group who used condoms were slightly (47.3%) above those from other ethnic groups, region. Youth(63.9%) who had multiple sexual partners were more likely to use condoms compared to those who had one sexual partner. Youth who ever tested for HIV (68.8%) used condoms than those who never tested for HIV. 69.0% of the youth belonging to protestant religion used condoms more than 12.9% from Muslim and other religions. Majority (59.2%) of the youth working used condoms more that those not working (16.6%).

**Table 4:** Bivariate analysis of the determinants of condom use among the youth in Malawi. Source: Malawi Demographic and Health Survey (2015/16), survey data

Independent variable	Male used a condom in the past 4 months			Female used a condom in the past 4 months			Condom use among the youth		
	Yes	No	Chi-square value (p-value)	Yes	No	Chi-square value (p-value)	Yes	No	Chi-square value (p-value)
	Y (%)	N (%)		Y (%)	N (%)		Y (%)	N (%)	
Age grouped									
15–19	394 (45.8)	200 (29.3)	43.416 (0.001)	518 (54.4)	1168(27.9)	248.027 (0.001)	912 (50.3)	1368 (28.1)	290.584 (0.001)
20–24	467 (54.2)	482 (70.7)		434 (45.6)	3023(72.1)		901 (49.7)	3505 (71.6)	
Marital status									
Never married	740 (85.9)	251 (36.8)	400.005 (0.001)	627 (65.9)	359 (8.6)	1643.438 (0.001)	1367 (75.4)	610 (12.5)	2508.86
Ever married	121 (14.1)	431 (63.2)		325 (34.1)	3832(91.4)		446 (24.6)	4263 (87.5)	-0.001
Place of residence									
Urban	212 (24.6)	95 (13.9)	27.302 (0.001)	276 (29.0)	750 (17.9)	59.811 (0.001)	488 (26.9)	845 (17.3)	75.915 (0.001)
Rural	649 (75.4)	587 (86.1)		676 (71.0)	3441(82.1)		1325(73.1)	4263 (87.5)	
Education									
No education	13 (1.5)	25 (3.7)	48.813 (0.001)	19 (2.0)	224 (5.3)	128.680 (0.001)	32 (1.8)	249 (5.1)	211. 942 (0.001)
Primary	469 (54.5)	470(68.9)		514 (54.0)	2875 (68.6)		983 (54.2)	3345 (68.8)	
Secondary and higher	379 (44.0)	187 (27.4)		419 (44.0)	1092 (26.1)		798 (44.0)	1279 (26.2)	
Religion									
Catholic	170 (19.7)	135 (19.8)	1.974 (0.373)	180 (16.7)	700 (16.7)	3.873 (0.144)	350 (19.3)	835 (17.1)	5.148 (0.76)
Protestants	583 (67.7)	445 (65.2)		668 (70.2)	2966 (70.8)		1251(69.0)	34,411(70.0)	
Muslim and others	108 (12.5)	102 (65.2)		104 (10.9)	525 (12.5)		212(11.7)	627 (12.9)	
Ethnic group									
Chewa	216 (30.3)	233 (34.2)	29.119 (0.001)	245 (25.7)	1391 (33.2)	60.927 (0.001)	506 (27.9)	1624 (33.3)	66.226 (0.001)
Tumbuka	97 (11.3)	48 (7.0)		97 (10.2)	406 (9.7)		194 (10.7)	454 (9.3)	
Lomwe	138 (16,0)	138 (20.2)		196 (20.6)	768 (18.3)		334 (18.4)	906 (18.6)	
Tonga	16 (2.3)	16 (2.3)		61 (6.4)	116 (2.8)		109 (6.0)	132 (2.7)	
Yao	92 (10.7)	80 (11.7)		112 (11.8)	496 (11.8)		204 (11.3)	576 (11.8)	
Sena	34 (5.0)	34 (5.0)		36 (4.6)	192 (4.6)		79 (4.4)	226 (4.6)	
Nkhonde	8 (1.2)	8 (1.2)		9 (0.9)	53(1.3)		30 (1.7)	61 (1.3)	
Ngoni	115 (13.4)	80 (11.7)		121 (12.7)	502 (12.0)		236 (13.0)	582 (11.9)	
Mang’anja	17 (2.5)	17 (2.5)		24 (2.6)	80 (1.6)		97 (2.0)	97 (2.0)	
Nyanya	8 (0.9)	6 (0.9)		30 (3.2)	66 (1.6)		72 (1.5)	72 (1.5)	
Others	17 (2.0)	22 (3.2)		21 (2,2)	121 (2.9)		143 (2.9)	142 (2.9)	
Region									
Northern	192 (22.3)	91 (13.3)	20.497 (0.001)	204 (21.4)	720 (17.9)	21.747 (0.001)	396 (21.8)	811 (16.6)	33.785 (0.001)
Central	279 (32.4)	252 (37.0)		280 (29.4)	1546 (36.9)		559 (30.8)	1798 (36.9)	
Southern	390 (45.3)	339 (49.7)		468 (49.2)	1925 (45.9)		585 (47.3)	2264 (46.5)	
Currently working									
No	237 (27.5)	113 (16.6)	26.052 (0.001)	498 (52.3)	1742 (41.6)	36.437 (0.001)	735 (40.5)	1855 (38.1)	3.407 (0.065)
Yes	624 (72.5)	569 (83.4)		454 (47.7)	2449 (58.4)		1078(59.5)	3018 (61.9)	
Lifetime number of sexual partners									
One sexual partner	175 (20.3)	116 (17.0)	2.735 (0.98)	480 (50.4)	2347 (56.6)	9.761 (0.002)	655 (36.1)	2463 (50.5)	110.342 (0.001)
Multiple sexual partners and others	686 (79.7)	566 (83.0)		472 (49.6)	1844 (44.0)		1158(63.9)	2410 (49.5)	
Ever tested for HIV									
No	312 (37.3)	204 (29.9)	9.209 (0.002)	245 (25.7)	418 (10.0)	171.624 (0.001)	56 (31.2)	622 (12.8)	308.004 (0.001)
Yes	540 (62.7)	478 (70.1)		707 (74.3)	3773 (90.0)		1247 (68.8)	251 (87.2)	

p-value less than 0.05 means variable is statistically significant

In terms of sex male aged 20–24 (54.2%) used condoms slightly above to those aged 15–19 (45.8%). Majority of the male who were not married(85.9%) used condoms more than their married counterparts, 75.4% of male residing in rural area used condoms more than 24.6% residing in urban area. Male with primary education (54.5%) who used condoms was slightly higher compared to the male youth with no education or those with secondary education or higher. The proportion of male youth from chewa ethnic group who used condoms was slightly (30.3%) higher than ethnic groups, region (45.3%). Majority of male who were currently working (72.5%) used condoms than those not working and the proportion of those who ever tested for HIV (62.7%) was higher than those who never tested for HIV. 67.7% male belonging to protestant religion used condoms more than 19.8% catholic men. Majority (79.9%) of male with multiple sexual partners used condoms than those with one sexual partner (17.0%).

Among the female those aged 15–19 (54.4%) used condoms slightly higher than 20–24 (45.6%), majority of female who were not married (65.9%) used condoms more than their married counterparts. Majority (71.0%) of female residing in rural areas used condoms more than those from the urban did. The proportion of the female who had primary education (54.0%) used condoms slightly higher than those with no education or those with secondary education or higher. The female youth from chewa ethnic group (25.7%) used condoms slightly higher than those from other ethnic groups did. Most (49.2%) of female from southern region used condoms higher than those from the northern and central region., working class female (52.3%) used condoms slightly higher than those who were not working (47.7%) The proportion of 50.4% female who had one sexual partner slightly used condoms higher than 49.6% with multiple sexual partners and those who ever tested for HIV (74.3%) used condoms more than the female who never tested for HIV. Majority (69.0%) of female Protestants used condoms than female (12.9%) Muslims and those who belong to other religions.

### Odds of condom use among the youth in Malawi

. The odds of condom use among the youth as presented in Table 5 were significantly lower in female (OR 0.39 CI 0.33–0.47) compared to male, youth aged 15–19 (OR 0.80 CI 0.68–0.95) compared to youth aged 20–24, never married (OR 0.07 CI 0.06–0.08) compared to their married counterparts, central (OR 0.56 CI 0.40–0.77) and southern regions (OR 0.59 CI 0.42–0.83) compared to those from the northern region as well as residence in a rural area (OR 0.74 CI 0.61–0.90) compared to those from urban areas. However, odds for those who had ever tested for HIV were significantly higher (OR 1.29 CI 1.03–1.55) compared to those who never tested for HIV.

**Table 5:** Odds of condom use among the youth in Malawi. Source: Malawi Demographic and Health Survey (2015/16), survey data, (Ref.)—Reference category, ***p < 0.001, *p < 0.05

Independent variable	Male	Female	All youth
	OR (95% CI)	OR (95% CI)	OR (95% CI)
Sex			
Male (Ref.)	1		
Female	0.39(0.33–0.47)***		
Age grouped			
15–19	1.30 (0.97–1.73)	0.61(0.50–0.75)***	0.80 (0.68- 0.95)***
20–24(Ref)	1	1	1
Marital Status			
Ever married (Ref.)	1	1	1
Never married	0.08 (0.06–0.11)***	0.06 (0.05–0.07)***	0.07 (0.06–0.08)***
Religion			
Muslim and others (Ref)	1	1	1
Catholic	0.91 (0.68–1.23)	0.90(0.72–1.13)	0.90 (0.75–1.07)
Protectants	0.92 (0.57–1.49)	1.06 (0.70–1.60)	0.97 (0.71–1.33)
Ethnic group			
Nyanja (Ref.)	1	1	1
Chewa	1.30 (0.71–2.39)	0.95 (0.62–1.45)	1.05 (0.75–1.48)
Tumbuka	0.91 (0.60–1.39)	1.11 (0.81–1.52)	1.07 (0.84–1.38)
Lomwe	1.61 (0.49–5.29)	1.35 (0.72–2.52)	1.38 (0.80–2.35)
Tonga	0.97 (0.58–1.62)	0.82 (0.55–1.23)	0.91 (0.66–1.25)
Yao	0.73 (0.37–1.42)	0.84 (0.48–1.48)	0.78 (0.51–1.20)
Sena	0.99 (0.30–3.25)	0.64 (0.20–2.03)	0.84 (0.39–1.84)
Nkhonde	0.77 (0.52–1.15)	0.98 (0.72–1.33)	0.90 (0.71–1.14)
Ngoni	1.06 (0.45–2.48)	1.65 (0.90–3.02)	1.48 (0.90–2.44)
Mang’anja	0.69 (0.06–7.99)	1.47 (0.65–3.33)	1.32 (0.61–2.86)
Others	0.48 (0.18–1.32)	1.32 (0.65–2.69)	0.91 (0.50–1.65)
Region			
Northern (Ref.)	1	1	1
Central	0.53 (0.30–0.93)***	0.58 (0.39–0.88)***	0.56 (0.40–0.77)***
Southern	0.55(0.30–0.98)***	0.64 (0.41–0.98)***	0.59 (0.42–0.83)***
Place of Residence			
Urban (Ref.)	1	1	1
Rural	0.66(0.46–0.94)***	0.78 (0.62–0.99)***	0.74 (0.61–0.90)***
Education			
No education (Ref.)	1	1	1
Primary	1.07 (0.51–2.23)	1.16 (0.70–1.92)	1.12 (0.74–1.70)
Secondary and higher	1.37 (0.64–2.92)	1.35(0.79–2.28)	1.39 (0.91–2.13)
Number of lifetime sexual partners			
One sexual partner (Ref)	1	1	1
Multiple sexual partners and others	0.93 (0.69–1.25)	1.18 (0.99–1.42)	1.11 (0.95–1.29)
Ever tested for HIV			
No (Ref.)	1	1	1
Yes	1.24 (0.95–1.62)	1.36 (1.07–1.74)***	1.29 (1.03–1.55)***
Currently working			
No (Ref.)	1	1	1
Yes	1.37 (1.01–1.85)*	1.04 (0.86–1.25)	1.13(0.97–1.32)

The odds of condom use among the male youth were significantly lower in those who were never married (OR 0.08 CI 0.06–0.11) compared to their married counterparts, central (OR 0.53 CI 0.30–0.93) and southern regions (OR 0.55 CI 0.30–0.98) compared to the youth from the northern region as well as residing in the rural area (OR 0.66 CI 0.46–0.94) compared to those residing in urban area. However, the odds for youth who were currently working were significantly higher (OR 1.37 CI 1.01–1.85) compared to those who were not working.

The odds of condom use among the female youth were to be significantly lower in female aged 15–19 (OR 0.61 CI 0.50–0.75) than those aged 20–24, never married (OR 0.06 CI 0.05–0.07) compared to their married counterparts, central (OR 0.58 CI 0.39–0.88), and southern regions (OR 0.64 CI 0.41–0.98) compared to those from northern region as well as residing in the rural area (OR 0.78 CI 0.62–0.99) compared to those residing in urban area. However, the odds of condom use among those who had ever tested for HIV were significantly higher (OR 1.36 CI 1.07–1.74) compared to those who had not tested for HIV.

## Discussion

The study examined the prevalence and determinants of condom use among sexually active youth in the last sexual intercourse within 4 months before the study. The results showed the prevalence of condom use to be higher among the male 861 (55.8%) than female 952 (18.5%). Similar results were also found in some studies conducted in Kenya and Benin [15, 28]. Low prevalence of condom use facilitates the spread of STI’s including HIV and unplanned pregnancies [2]. The vulnerability of women affects their negotiation power to safer sex unless a man initiates condom use. On the other hand male condoms are easily accessible than female condoms as such it is easier for men to have control over their use it than women. However, some studies conducted in Cambodia, Ethiopia, and Ghana showed a high prevalence of condom use among female[17, 29, 30]. The difference in the findings could be due to socio-economic status, availability of condoms, carrier aspirations, and expectations of the youth [17]. In the current study, the highest level of education for about 69% of the youth was no education and primary, indicating low socio-economic status.

Among all the youth low prevalence of 27.1% for condom use was established during the last sexual intercourse within 4 months before the study. These findings were similar to a study conducted by Perera and Abeysena (2018) in Sri Lanka who found that 85.8% of sexually active undergraduates did not use condoms in their last sexual activity. Sex distribution in the study population explains why the percentage was very low since female youth consisted of the majority of the study population. On the contrary, Nesidai and colleagues in Kenya established the prevalence of condom use to be high among the youth 72.8% [2]. The results were expected to be similar to Malawi because the government and other stakeholders provide condoms free. Since some literature has shown that providing free condoms increases the level of condom use [31].. This calls for an assessment of the effectiveness of the interventions responding to condom use among the youth in Malawi.

Regarding the determinants of condom use among the youth in Malawi, results revealed some gender differences. The study established male youth to be more likely to use condoms than female youth in their last sexual encounter within 4 months before the study. The results were similar to research done in the United States, Kenya, and Benin [15, 28, 32]. These results were due to a lack of negotiation skills for protected sex, unavailability of female condoms, not being comfortable to talk about sex with a partner, and having insufficient knowledge on condom use and its importance among the female youth [15].

A difference in age range among male and female youth was noted, as younger male (15–19 years) were insignificantly more likely to use a condom while female of same age were significantly less likely to use condom. The results were contrary to a study carried out in Kenya which found that the majority of older youth (20–24 years) were more likely to use condoms and similarly with female aged 15–19 [2]. In both cases, the results may be because of differences age distribution of the study population. In addition, not wanting to become pregnant or getting someone pregnant at a tender age might have attributed to the results.

Marital status was another variable taken into consideration. The study established among all the male and female youth those who were ever married were more likely to practice safe sex than those were never married. The findings are similar to a study conducted in Brazil [31] but differ with Nesidai and colleagues in Kenya who found no significant results on marital status[2]. As far as population distribution might have contributed to the results. The other reason could be that never married male and female youth may engage in transaction sex in exchange of material goods or other benefits[33, 34].

In terms of place of residence, the study found that male and female youth residing in a rural area had a decreased likelihood of using condoms in their last sexual intercourse within 4 months before the study. On contrary, a study by Sabegeh, place of residence did not produce significant results [13]. The reason for the contrary results was due to population distribution in the place of residence. In Malawi, majority (86%) of the population resides in rural areas which are characterized by insufficient knowledge on condom use and services..

Education level was also given consideration and among all the youth education was found not significant. The results differ from other studies conducted in Malawi[16, 35]. Education status was expected to be significant as in the past studies because the Malawian government-owned schools offer free education, which gives room for every child to have access to at least primary education. Education equips with different knowledge including on STI's including HIV and unplanned pregnancies and this has the ability to empower the youth to decide whether to practice safe sex or not [2, 35].

On working status, currently, working male were more likely to practice safe. On the contrary, the female who were working were more likely to practice safe, but insignificantly so.. However, the study did not produce the results as expected because working-class males often engage in multiple relationships and some studies found this often leads to unprotected sexual intercourse [16]. For the female, it was expected female who are currently working to more likely to use condoms because of being empowered and able to negotiate for safer sex [2].

Region and ethnic group was also taken into consideration. There was no significant result in ethnic group. On region, youth whether male or female in the central and southern regions were significantly less likely to use condom compared to those in the Northern region. The results were attributed by population distribution as majority of the study population were from these region.

A “lifetime number of sexual partners”[27] was not significant in the study the result was contrary to other studies that found it to be significant [16]. Despite these findings, it was expected that an individual with multiple partners often use condoms to prevent STI's including HIV.

Among all the youth, those who have ever tested for HIV were more likely to use condoms. The reason is to protect themselves from contracting the virus in instances where the youth is tested negative. However, this is not always the case when one is tested positive some youth start engaging in unprotected sex to spread the virus. A study conducted in Brazil found a negative association on condom use for female youth who tested for HIV [31].

Concerning sex, the odds significantly higher for female who had ever tested for of HIV. The results have implications for the development of policies and programs regarding condom use among the youths. Intensifying HIV testing among the youth and the general public can promote the practicing of safe sex thereby preventing STI's including HIV and unplanned pregnancies. In addition, education should be emphasized among all the youths to empower them to make the right decisions about condom use.

## Study limitations

The study was limited to the variables recorded by DHS of 2015–2016 only. The study was cross-section which was the reflection of the state of the things at the time data was collected, much may have changed by now if a similar study is conducted. Furthermore, the responses presented depended on the respondent's ability to recall if he or she practiced safe sex within four months before the study as such this could lead to under or over-reporting hence affecting the results to some extent.

## Recommendation

The study recommends the following.

- Scaling up campaigns that will help to equip knowledge among the youth on the prevention of STI's including HIV and prevention of unplanned pregnancy through the use of a condom.
- Government and stakeholders should consider gender at the center of developing all programs and policies.
- Make Female condoms accessible and affordable to the general public.
- Promotion of sexual and reproductive health communication with the household which can assist the youth in the prevention of STI's including HIV and unplanned pregnancies.
- Scaling up the circulation of sexual and reproductive health information in other places where youth are found like church gatherings and community-based meetings.
- The study recommends an in-depth study on other determinants of condom use among the youth in Malawi which has not been looked at in this study but has been researched in other countries.

## Conclusion

Safe sex among the youth can contribute to an HIV-free nation and reduce unplanned pregnancies. Majority of sexually active youth in Malawi just as the majority of the youth in the world practice unsafe sex. Using the 2015–2016 Malawi Demographic Health Survey study established the prevalence of condom use among the youth in the past four months before the study to be very low. In regards to sex, the majority of male used more condoms than female in their last sexual encounter. The significant determinants of condom use among the youth in Malawi were: age between 15 and 19, never married, residing in rural, Chewa ethnic group, from the southern region of Malawi, and ever tested for HIV. In regards to sex, the significant determinants for male were: age 20–25, never married, and residing in rural, Chewa ethnic group, from the southern region of Malawi, currently working and ever tested for HIV. For the female, the determinants were: age 15–19, ever married, residing in rural, Chewa ethnic group, from the southern region of Malawi, currently not working and ever tested for HIV. Scaling up programs and developing policies that target the youth to practice safe sex will assist in addressing challenges of STI’s including HIV and preventing unplanned pregnancies.

## Data Availability

The data sets used for the analysis of this study are available for free from https://www.dhsprogram.com/data/dataset_admin/login_main.cfm after obtaining permission from ICF Micro International.

## Abbreviations

AIDS: Acquired Immunodeficiency Syndrome
CERHI: Center of Excellence in Reproductive Health Innovation
DHS: Demographic Health Survey
HIV: Human Immunodeficiency Virus
MDHS: Malawi Demographic Health Survey
STIs: Sexually transmitted infections
SPSS: Statistical Package for Social Sciences
UNAIDS: United Nations program on HIV and AIDS
